# Balancing act: exploring the impact of work–family conflict on anxiety among working parents with family health as a mediator

**DOI:** 10.3389/fpsyg.2025.1531091

**Published:** 2025-07-18

**Authors:** Chaochan Su, Meng Yang, Qiuxia Huang, Min Yang

**Affiliations:** ^1^The Second Affiliated Hospital, Guangzhou Medical University, Guangzhou, Guangdong, China; ^2^Guangzhou Nansha District Center for Disease Control and Prevention, Guangzhou, Guangdong, China; ^3^Guangdong Province Hospital for Occupational Disease Prevention and Treatment, Guangzhou, Guangdong, China

**Keywords:** work–family conflict, anxiety, family health, mediation, cross-sectional survey

## Abstract

**Objective:**

The increasing prevalence of work–family conflict and anxiety among working parents, particularly in China, underscores the importance of understanding their interrelationship. This study sought to investigate the relationship between work–family conflict (WFC) and anxiety among working parents while exploring the mediating role of family health in this relationship.

**Methods:**

A large-scale cross-sectional survey was conducted using data from the 2021 Psychology and Behavior Investigation of Chinese Residents. The sample comprised 5,068 occupational parents who met the research criteria. Anxiety was assessed using the 7-item Generalized Anxiety Disorder scale, WFC was measured using the WFC scale, and family health was evaluated using the Short Form of the Family Health Scale. Multinomial logistic regression and mediation analyses were applied to examine relationships.

**Results:**

Among the participants, 58.4% reported no symptoms of anxiety, 30.6% experienced mild anxiety, and 11.0% reported moderate to severe anxiety. Scores for WFC and family health demonstrated significant associations with anxiety levels. Specifically, higher levels of WFC were associated with an increased risk of mild anxiety (odds ratio [OR] = 1.058, 95% confidence interval [CI]: 1.051–1.064) and moderate to severe anxiety (OR = 1.123, 95% CI: 1.111–1.135). Conversely, higher family health scores were associated with a decreased risk of mild anxiety (OR = 0.934, 95% CI: 0.924–0.945) and moderate to severe anxiety (OR = 0.859, 95% CI: 0.842–0.876). Mediation analysis revealed that family health significantly mediated the relationship between WFC and anxiety levels (*p* < 0.05).

**Conclusion:**

The findings confirm a significant relationship between WFC and anxiety, with family health serving as a partial mediator. These results suggest that improving family health may represent an effective strategy for reducing anxiety among working parents.

## 1 Introduction

Anxiety is a prevalent mental health concern among working populations, with its incidence increasing recently (Holmgren et al., 2023; Hogg et al., 2021), particularly among employees in China (Li et al., 2022; Liang et al., 2025), highlighting the growing mental health challenges in the country. Several studies have revealed that the anxiety rate has risen as high as 30.2–47.12% across different industries (Cai et al., 2025; Liang et al., 2025; Lu et al., 2024). This upward trend is particularly pronounced among working parents, who encounter the distinct challenge of balancing occupational demands with family responsibilities (Perry-Jenkins et al., 2017). Studies have identified multiple factors influencing anxiety levels in working parents, including gender, age, shift work, and work-life balance (Sala et al., 2023; Torquati et al., 2019). Anxiety can adversely impact individual work performance and overall quality of life, and it may also contribute to severe mental health issues, such as physiological dysfunction and heightened suicidal ideation (Chalmers et al., 2021).

In the Chinese cultural context, anxiety not only affects individuals’ mental well-being but also has significant implications for family dynamics and work productivity (Annink et al., 2016). The traditional emphasis on collectivism and family harmony places additional pressures on working parents to excel in both professional and domestic domains. This cultural backdrop may exacerbate the impact of work–family conflict on anxiety (Wong and O’Driscoll, 2018). Furthermore, utilization of health services is low and delayed among individuals with anxiety disorders, despite high disease burdens and available effective treatments. The data from China Mental Health Survey revealed the failure and delays in help-seeking are common in China (Zhong et al., 2025), highlighting the need for community-based interventions to address this growing concern.

The acceleration of the working pace and the intensification of occupational pressure in contemporary society have increasingly blurred the boundaries between professional and personal life (Wandschneider et al., 2022), rendering work–family conflict (WFC) an escalating social concern (Hao et al., 2023; Bu et al., 2024). WFC refers to the interference and conflict between professional and familial roles, which can profoundly influence individual mental health (Haslam et al., 2015). Research has identified contributing factors to this conflict from both workplace and family perspectives, including the number of employees, occupational demands, divorce rates, household consumption levels, elderly dependency ratios, and family size (Xin et al., 2020). Moreover, the growing prevalence of remote work and flexible working hours has contributed to the increasing incidence of WFC (Wang, 2022), potentially intensifying the problem. Studies have demonstrated a significant positive correlation between WFC and anxiety, indicating that higher levels of WFC may contribute to increased anxiety among employees, particularly working parents (Wang et al., 2023; Xu et al., 2023). Anxiety may also exacerbate WFC reciprocally, creating a vicious cycle (Zhang et al., 2023; Adedeji et al., 2023).

Family health, as a critical component of overall individual health, may function as a protective factor against the adverse effects of WFC on anxiety (Arik Tasyikan and Demiral, 2022). It encompasses the physical health of family members, mental well-being, and the quality of familial relationships (Killien, 2004). Research suggests that a high level of WFC can strain family relationships and reduce emotional support, thereby deteriorating family health (Sobol and Ben-Shlomo, 2019). A harmonious family environment can provide a restorative space for working parents to recover from work-related pressures (Chen et al., 2022). The Conservation of Resources (COR) theory (Hobfoll, 1989) further explains that family health can help individuals conserve and allocate resources more efficiently, thereby mitigating the impact of WFC on anxiety. According to this theory, individuals are motivated to preserve valued resources; however, when these efforts are unsuccessful, psychological issues such as anxiety may arise due to a perceived inability to manage high demands. Resources tension is the foundation for role conflicts, as involvement in a specific role can intensify interference among multiple roles due to competition for limited resources (Hobfoll et al., 2018). WFC can increase stress and reduce the time and energy available for family activities, impairing family health. This decline in family health may exacerbate anxiety among working parents, as the basic motivation to preserve resources is compromised. Existing literature has also identified that WFC reduces employees’ sense of control and predicts a heightened risk of mental health disorders (Perry-Jenkins and Gerstel, 2020). Therefore, understanding the mediating role of family health is essential for designing effective interventions that address the interplay between work and family dynamics.

Although previous research has examined the associations among WFC, family health, and anxiety, notable gaps remain in the existing literature. While some studies have investigated the direct effect of WFC on anxiety (Xin et al., 2020), limited attention has been given to the mediating role of family health within this relationship. The relationship between WFC and anxiety may vary across working individuals with different occupational characteristics, cultural backgrounds, and family structures; however, research in this area remains relatively limited. Additionally, identifying strategies to alleviate anxiety in working parents by improving family health and designing effective intervention approaches warrants further scholarly attention (Cavagnis et al., 2023).

This study aimed to elucidate the mediating role of family health in the relationship between WFC and anxiety, providing insights into how family dynamics can influence mental health outcomes among working parents. By investigating this relationship, this study highlights the importance of family health as an essential element of overall well-being. It aims to identify potential strategies for reducing anxiety among working parents. The findings provide a novel perspective for understanding mental health challenges among working parents and provide scientific evidence for developing effective mental health promotion strategies. Based on the COR theory framework, the following hypotheses were formulated to guide the present study:

*H1*: WFC has a significant negative impact on family health.

*H2*: Family health has a significant adverse predictive effect on anxiety, such that higher levels of family health are associated with lower levels of anxiety.

*H3*: WFC is a significant positive predictor of anxiety, suggesting that higher levels of WFC correspond to increased anxiety levels.

*H4*: Family health serves as a partial mediator in the relationship between WFC and anxiety.

## 2 Methods

### 2.1 Participants and data collection

The data utilized in this study were derived from the 2021 Psychology and Behavior Investigation of Chinese Residents. The survey initiative was designed to construct a database through a multicenter, large-scale, and cross-sectional study. Its objective was to provide robust data support for advancing research across multiple disciplines and to facilitate a comprehensive and systematic understanding of the physical and mental status of the general population.

The investigation encompassed 23 provinces, 5 autonomous regions, and 4 municipalities directly administered by the central government between July and September 2021. The survey adopted a multi-stage sampling method, employing the random number table technique to select 2–6 cities from each noncapital prefecture-level administrative region within each province and an autonomous region. A total of 120 cities were incorporated in the study. Based on the “2021 Seventh National Census” data, quota sampling was implemented across 120 cities, with gender, age, and urban–rural distribution serving as quota attributes so that the gender, age, and urban–rural distribution of the samples generally conformed to the demographic characteristics. For data collection, each city designated either an individual surveyor or a team of surveyors to distribute and retrieve questionnaires. Individual surveyors were assigned to collect between 30 and 90 questionnaires, whereas teams targeted a collection range of 100–200 questionnaires. The surveyor utilized the Online Questionnaire Star platform^1^ for data collection and obtained informed consent from each participant. A total of 11,031 valid questionnaires were collected, all meeting high-quality standards and adhering to ethical review guidelines (He et al., 2022).

The inclusion criteria for the sample in this study were as follows: (1) Individuals aged 18 years or older; (2) nationality of the People’s Republic of China; (3) residency of China (with an annual absence from home not exceeding 1 month); (4) working population, including individuals employed full-time or with no fixed employment; (5) having one or more children. The exclusion criteria were as follows: (1) Students; (2) retirees; (3) individuals without children; (4) individuals unwilling to participate; (5) missing key variables.

**Figure 1:**
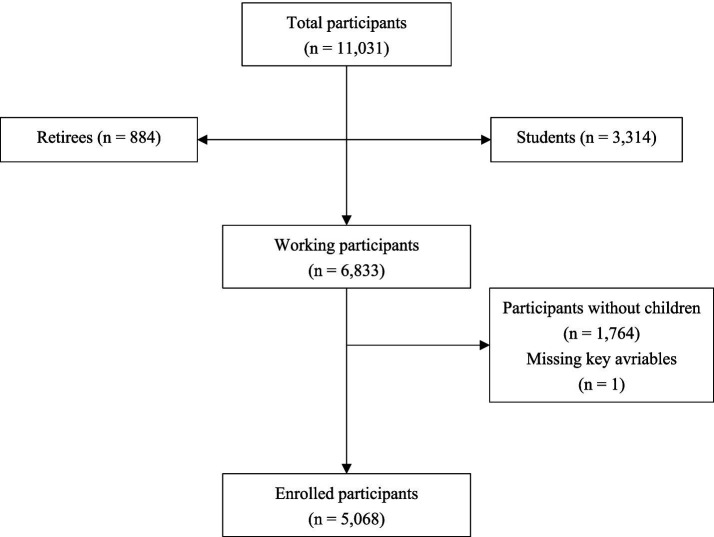
Flowchart of participant selection.

Based on the sample inclusion and exclusion criteria, research data from 5,068 working parents were ultimately included in this study (Figure 1).

### 2.2 Measures

*The 7-item Generalized Anxiety Disorder Scale* (GAD-7), developed by Spitzer et al. (2006), has become widely utilized in clinical settings and various studies in China (Huang et al., 2023), particularly for screening anxiety disorders. The GAD-7 comprises seven items that assess symptoms of generalized anxiety, such as “feeling tense, anxious, or on edge” and “unable to stop or control worrying.” Participants were asked to report the frequency of these symptoms over the past 2 weeks based on their experience. The scale was scored on a four-point range, with responses from “0 = not at all” to “3 = almost every day” (Cronbach’s *α* = 0.946). Higher scores on the scale correspond to greater anxiety severity. A total score of 0–4 indicates no anxiety disorder, 5–9 suggests mild anxiety, and a score above 10 reflects moderate and severe anxiety.

*The Work–Family Conflict Scale* (WAFCS) (Haslam et al., 2015) comprises two dimensions: work-to-family conflict (items such as “work frequently makes me irritable or short-tempered at home”) and family-to-work conflict (items such as “family-related concerns or responsibilities often distract me at work”). Each dimension includes five items, rated on a 7-point Likert scale from 1 (very strongly disagree) to 7 (very strongly agree). Scores from each dimension were calculated by summing the respective items, and the total score was determined based on the sum of each dimension’s points. Higher scores indicate greater levels of WFC. The scale has been translated into Chinese and applied across different fields (Hao et al., 2023). Cronbach’s alpha for this study was 0.945.

*The Short Form of the Family Health Scale* (Crandall et al., 2020) was employed to assess family health. The Chinese version of the Short Form of the Family Health Scale has demonstrated good reliability and validity. It has been utilized to evaluate the level of family health among Chinese residents (Wang et al., 2022). Items such as “We help each other and make healthy changes” were adapted to align with Chinese linguistic habits while preserving the original meaning. The scale contains four dimensions: (1) Family social or emotional health processes; (2) family healthy lifestyle; (3) family health resources; (4) family external social support. Scores on the scale ranged from 10 to 50, with a higher score indicating better family health. In the present study, Cronbach’s alpha was 0.745, reflecting acceptable reliability.

### 2.3 Covariates

The study incorporated several key covariates in the questionnaire to assess the influence of potential confounding factors. These factors included sex, age, race, educational attainment, marital status, occupational status, family structure, household income, number of children, number of illnesses, and medical insurance.

### 2.4 Statistical analysis

Participants were categorized into three groups based on their level of anxiety. Differences in baseline variables were examined using Chi-square tests. This study treated the anxiety level variable as an ordinal categorical variable. However, the assumption of parallel lines required for ordinal logistic regression analysis was not satisfied, making this method inappropriate. Consequently, multinomial logistic regression was employed to analyze the effect of WFC and family health on anxiety. Model 1 included marital status and number of children; Model 2 incorporated family structure, income, occupational status, number of diseases, and medical insurance; Model 3 further incorporated gender, age, race, and educational attainment. Odds ratios (ORs) and 95% confidence intervals (CIs) were calculated to estimate the risk of anxiety.

The PROCESS Macro (Model 4) with bootstrapping was employed to estimate direct and indirect effects and investigate the mediating role of family health. A total of 5,000 bootstrap samples were used in this study. This approach was applied to determine whether family health mitigated the effect of WFC on anxiety. Restricted cubic splines (RCS) were also utilized to assess the potential non-linear relationship between WFC and anxiety. Using RCS allowed for a more detailed understanding of how WFC associates with anxiety risk across varying ranges, potentially revealing thresholds or inflection points for association. Finally, sensitivity analyses were conducted using multiple linear regression and subgroup analyses stratified by gender to verify the robustness of our findings and explore potential gender differences. All statistical analyses were performed using Statistical Package for the Social Sciences (SPSS, version 22.0), PROCESS Macro (version 4.1), and R (version 3.6.3) software, with statistical significance set at *p* < 0.05.

## 3 Results

### 3.1 Participant demographics

A total of 5,068 individuals were surveyed, with 2,960 (58.4%) reporting no symptoms of anxiety. A total of 1,550 participants (30.6%) experienced mild anxiety, while 558 (11.0%) reported moderate to severe anxiety. These findings suggest a progressive increase in anxiety prevalence within the occupational population, highlighting its emergence as a significant mental health concern. Table 1 presents the variations in anxiety distribution across groups with different demographic characteristics. Among participants from core families, 60.68% reported a higher proportion of “no anxiety,” while the proportion experiencing moderate to severe anxiety (8.94%) was lower compared to the other types of families, with statistically significant differences (*p* < 0.01). Core families, defined as a married couple and unmarried children, represent the traditional family structure in China. Respondents with the lowest family income (less than 3,000 yuan per month) reported a higher incidence of anxiety compared to other groups (*p* < 0.05). The prevalence of anxiety among females (43.25%) was higher than that of males, with the majority experiencing mild anxiety (32.84%), a statistically significant difference (*p* < 0.01). Among the various age groups within the surveyed population, the individuals under the age of 35 exhibited a significantly higher proportion of moderate to severe anxiety (14.38%) than those in other age groups (8.34–11.66%) (*p* < 0.01). Younger parents are required to dedicate more energy to caring for underage children at home. Consequently, when work-life conflicts occur, they are more prone to experiencing anxiety. A statistically non-significant difference was observed in the proportion of respondents with varying levels of anxiety across different education levels (*p* > 0.05). Among individuals with different marital statuses, the proportion of singles, including those who are unmarried, divorced, or widowed, experiencing anxiety was significantly higher than that of married individuals. The rates of mild and moderate to severe anxiety were 33.44 and 19.50% in single/divorced/widow group, respectively, compared to 30.39 and 10.43% in the married group. As the number of children increased, the proportion of parents experiencing anxiety also gradually increased. Among participants with three or more children, 33.98% experienced severe anxiety, highlighting the need for employers to consider the family dynamics of employees with many children in their daily management. Additionally, the impact of different occupational statuses on anxiety revealed significant differences. Relatively high rates of mild anxiety and moderate to severe anxiety were observed in the irregular work group compared to full-time employees. Regarding personal health and medical insurance, individuals with a higher number of health conditions tend to experience greater levels of anxiety. Among respondents without health insurance, the proportion of moderate to severe anxiety was 1.7 times greater (17.30%) than that of the insured group (10.02%) (Table 1).

**Table 1:** Demographic characteristics and occupational status of participants.

Variables	Total (*n* = 5,068)	No anxiety (*n* = 2,960)	Mild anxiety (*n* = 1,550)	Moderate/severe anxiety (*n* = 558)	Statistic	*p*-value
Family structure, *n* (%)					*χ*^2^ = 38.37	<0.001
Other family type	1,968 (38.83)	1,079 (54.83)	608 (30.89)	281 (14.28)		
Core family	3,100 (61.17)	1,881 (60.68)	942 (30.39)	277 (8.94)		
Family income, *n* (%)					*χ*^2^ = 15.80	0.015
<3,000	1,492 (29.44)	820 (54.96)	487 (32.64)	185 (12.40)		
3,000–	2,007 (39.60)	1,192 (59.39)	621 (30.94)	194 (9.67)		
6,000–	1,241 (24.49)	752 (60.60)	348 (28.04)	141 (11.36)		
12,000–	328 (6.47)	196 (59.76)	94 (28.66)	38 (11.59)		
Sex, *n* (%)					*χ*^2^ = 14.48	<0.001
Female	2,719 (53.65)	1,543 (56.75)	893 (32.84)	283 (10.41)		
Male	2,349 (46.35)	1,417 (60.32)	657 (27.97)	275 (11.71)		
Age group (year), *n* (%)					*χ*^2^ = 29.26	<0.001
<35	876 (17.28)	488 (55.71)	262 (29.91)	126 (14.38)		
35–	1,838 (36.27)	1,050 (57.13)	574 (31.23)	214 (11.64)		
45–	1,702 (33.58)	1,058 (62.16)	502 (29.49)	142 (8.34)		
55–	652 (12.87)	364 (55.83)	212 (32.52)	76 (11.66)		
Race, *n* (%)					*χ*^2^ = 14.35	<0.001
Han	4,772 (94.16)	2,816 (59.01)	1,445 (30.28)	511 (10.71)		
Other	296 (5.84)	144 (48.65)	105 (35.47)	47 (15.88)		
Education, *n* (%)					*χ*^2^ = 6.62	0.358
Junior or less	1,676 (33.07)	942 (56.21)	544 (32.46)	190 (11.34)		
High school	1,037 (20.46)	620 (59.79)	300 (28.93)	117 (11.28)		
Junior college	823 (16.24)	487 (59.17)	254 (30.86)	82 (9.96)		
Bachelor or more	1,532 (30.23)	911 (59.46)	452 (29.50)	169 (11.03)		
Marital status, *n* (%)					*χ*^2^ = 31.13	<0.001
Single/divorced/widow	323 (6.37)	152 (47.06)	108 (33.44)	63 (19.50)		
Married	4,745 (93.63)	2,808 (59.18)	1,442 (30.39)	495 (10.43)		
Child number, *n* (%)					*χ*^2^ = 17.54	0.002
1	2,696 (53.21)	1,623 (60.18)	269 (10.01)	804 (29.81)		
2	1,910 (37.68)	1,101 (57.64)	220 (11.52)	589 (30.84)		
3 or more	462 (9.11)	236 (51.08)	69 (14.94)	157 (33.98)		
Occupational status, *n* (%)					*χ*^2^ = 24.14	<0.001
Employed full time	3,286 (64.84)	1,999 (60.83)	958 (29.15)	329 (10.01)		
No fixed employment	1,782 (35.16)	961 (53.93)	592 (33.22)	229 (12.85)		
Sickness number, *n* (%)					*χ*^2^ = 63.61	<0.001
0	3,802 (75.02)	2,335 (61.42)	386 (10.15)	1,081 (28.43)		
1	871 (17.19)	450 (51.66)	109 (12.51)	312 (35.82)		
2 or more	395 (7.79)	175 (44.30)	63 (15.95)	157 (39.75)		
Medical insurance, *n* (%)					*χ*^2^ = 45.81	<0.001
Covered	4,380 (86.42)	2,627 (59.98)	1,314 (30.00)	439 (10.02)		
Self-paying	688 (13.58)	333 (48.40)	236 (34.30)	119 (17.30)		

*χ*^2^, Chi-square test.

### 3.2 Impact of WFC and family health on anxiety

Multinomial logistic regression results indicated that WFC and family health exerted statistically significant influences on anxiety levels (*p* < 0.001) but in opposing directions. In model 1, higher WFC scores were significantly associated with an increased risk of experiencing mild (OR = 1.055, 95% CI: 1.049–1.061) and moderate to severe anxiety (OR = 1.121, 95% CI: 1.110–1.133). Conversely, higher scores on family health were significantly associated with reduced risk of mild (OR = 0.937, 95% CI: 0.927–0.947) and moderate to severe anxiety (OR = 0.859, 95% CI: 0.843–0.875). Model 1 included two variables (marital status and number of children) susceptible to WFC. The findings revealed that married participants exhibited a significantly lower risk of developing mild (OR = 0.714, 95% CI: 0.541–0.941) and moderate to severe anxiety (OR = 0.440, 95% CI: 0.303–0.638) compared to those who were single, divorced, and widowed. An increase in the number of children was associated with a heightened risk of anxiety, particularly among parents with three or more children. In this group, the risk of experiencing mild (OR = 1.582, 95% CI: 1.247–2.006) and moderate to severe anxiety (OR = 2.427, 95% CI: 1.712–3.440) increased significantly, with the difference reaching statistical significance (*p* < 0.01).

Additional variables were incorporated into the regression model to assess the influence of these factors on the aforementioned association. Model 2 added several variables, including family structure, income, occupational status, existing health conditions, and medical insurance coverage. The regression model revealed that the effects of WFC and family health on anxiety remained statistically significant (*p* < 0.001), maintaining the same directional trend observed in Model 1. Specifically, higher levels of WFC significantly increased the risk of mild (OR = 1.056, 95% CI: 1.050–1.062) and moderate to severe anxiety (OR = 1.122, 95% CI: 1.111–1.134). Conversely, higher family health scores significantly reduced the risk of mild (OR = 0.936, 95% CI: 0.926–0.946) and moderate to severe anxiety (OR = 0.859, 95% CI: 0.843–0.876). Participants with two or more diseases exhibited a significantly increased risk of mild anxiety (OR = 2.036, 95% CI: 1.592–2.604) and moderate to severe anxiety (OR = 2.441, 95% CI: 1.692–3.522) relative to those without any diseases. Compared to individuals in full-time employment, those without fixed occupational status experienced a significantly higher risk of mild (OR = 1.214, 95% CI: 1.035–1.425) and moderate to severe anxiety (OR = 1.330, 95% CI: 1.039–1.703) (*p* < 0.05). Among additional variables included in Model 2, single marital status and having three or more children remained significantly associated with an increased risk of moderate to severe anxiety (*p* < 0.05).

In Model 3, demographic characteristics variables such as gender and age were incorporated into the regression model. The effect of WFC and family health on anxiety remained statistically significant (*p* < 0.001), with consistent direction patterns observed in Model 1 and Model 2. Higher WFC significantly increased the risk of mild (OR = 1.058, 95% CI: 1.051–1.064) and moderate to severe anxiety (OR = 1.123, 95% CI: 1.111–1.135), whereas higher family health scores significantly reduced the risk of mild (OR = 0.934, 95% CI: 0.924–0.945) and moderate to severe anxiety (OR = 0.859, 95% CI: 0.842–0.876). Among the other variables, the number of diseases and gender demonstrated significant associations with the risk of anxiety (*p* < 0.05). Male participants exhibited a significantly lower risk of mild (OR = 0.657, 95% CI: 0.573–0.754) and moderate to severe anxiety (OR = 0.752, 95% CI: 0.608–0.930) (Table 2).

**Table 2:** Multinomial logistic regression analysis of the association between WFC, family health, and anxiety.

Predictor variables	Model 1		Model 2		Model 3	
	OR (95% CI)	*p*-value	OR (95% CI)	*p*-value	OR (95% CI)	*p*-value
**Mild anxiety (ref: no anxiety)**						
Intercept	1.384 (0.808–2.371)	0.236	1.067 (0.606–1.876)	0.823	1.18 (0.654–2.127)	0.583
Family health	0.937 (0.927–0.947)	<0.001	0.936 (0.926–0.946)	<0.001	0.934 (0.924–0.945)	<0.001
WFC	1.055 (1.049–1.061)	<0.001	1.056 (1.05–1.062)	<0.001	1.058 (1.051–1.064)	<0.001
Child number (ref: 1)						
2	1.022 (0.889–1.176)	0.759	0.948 (0.819–1.098)	0.479	0.945 (0.815–1.096)	0.454
3 or more	1.582 (1.247–2.006)	<0.001	1.27 (0.983–1.642)	0.068	1.228 (0.942–1.6)	0.129
Marital status (ref: single/divorced/widow)						
Married	0.714 (0.541–0.941)	0.017	0.78 (0.586–1.038)	0.088	0.839 (0.629–1.118)	0.23
Family type (ref: other family type)						
Core family	N/A	N/A	1.091 (0.947–1.257)	0.226	1.104 (0.95–1.284)	0.197
Family income (ref: <3,000)						
3,000–	N/A	N/A	0.943 (0.797–1.115)	0.492	0.948 (0.8–1.122)	0.533
6,000–	N/A	N/A	0.979 (0.804–1.191)	0.829	0.999 (0.819–1.217)	0.988
12,000–	N/A	N/A	1.053 (0.778–1.426)	0.736	1.087 (0.801–1.474)	0.592
Occupational status (ref: Employed full time)						
No fixed employment	N/A	N/A	1.214 (1.035–1.425)	0.018	1.175 (0.996–1.387)	0.055
Sickness number (ref: 0)						
1	N/A	N/A	1.506 (1.264–1.795)	<0.001	1.581 (1.316–1.898)	<0.001
2 or more	N/A	N/A	2.036 (1.592–2.604)	<0.001	2.201 (1.701–2.849)	<0.001
Medical insurance (ref: covered)						
Self-paying	N/A	N/A	1.071 (0.877–1.307)	0.504	1.062 (0.868–1.298)	0.559
Sex (ref: female)						
Male	N/A	N/A	N/A	N/A	0.657 (0.573–0.754)	<0.001
Age (ref: <35 years)						
35–	N/A	N/A	N/A	N/A	1.047 (0.857–1.278)	0.655
45–	N/A	N/A	N/A	N/A	0.952 (0.774–1.171)	0.641
55–	N/A	N/A	N/A	N/A	1.047 (0.786–1.395)	0.752
Race (ref: Han)						
Minority	N/A	N/A	N/A	N/A	1.271 (0.957–1.689)	0.098
**Moderate to severe anxiety (ref: no anxiety)**						
Intercept	1.259 (0.547–2.899)	0.588	0.86 (0.359–2.065)	0.736	1.044 (0.424–2.574)	0.925
Family health	0.859 (0.843–0.875)	<0.001	0.859 (0.843–0.876)	<0.001	0.859 (0.842–0.876)	<0.001
WFC	1.121 (1.11–1.133)	<0.001	1.122 (1.111–1.134)	<0.001	1.123 (1.111–1.135)	<0.001
Child number (ref: 1)						
2	1.132 (0.908–1.411)	0.27	1.049 (0.834–1.32)	0.683	1.053 (0.835–1.328)	0.665
3 or more	2.427 (1.712–3.44)	<0.001	1.851 (1.274–2.688)	0.001	1.777 (1.21–2.609)	0.003
Marital status (ref: single/divorced/widow)						
Married	0.44 (0.303–0.638)	<0.001	0.534 (0.364–0.783)	0.001	0.56 (0.381–0.825)	0.003
Family type (ref: other family type)						
Core family	N/A	N/A	0.848 (0.683–1.051)	0.132	0.887 (0.709–1.111)	0.297
Family income (ref: <3,000)						
3,000–	N/A	N/A	0.852 (0.655–1.109)	0.233	0.864 (0.664–1.126)	0.28
6,000–	N/A	N/A	1.238 (0.916–1.675)	0.165	1.27 (0.937–1.72)	0.123
12,000–	N/A	N/A	1.28 (0.804–2.037)	0.298	1.313 (0.824–2.093)	0.252
Occupational status (ref: Employed full time)						
No fixed employment	N/A	N/A	1.33 (1.039–1.703)	0.024	1.31 (1.016–1.69)	0.037
Sickness number (ref: 0)						
1	N/A	N/A	1.407 (1.071–1.849)	0.014	1.534 (1.153–2.041)	0.003
2 or more	N/A	N/A	2.441 (1.692–3.522)	<0.001	2.715 (1.846–3.992)	<0.001
Medical insurance (ref: covered)						
Self-paying	N/A	N/A	1.274 (0.963–1.686)	0.089	1.262 (0.953–1.671)	0.104
Sex (ref: female)						
Male	N/A	N/A	N/A	N/A	0.752 (0.608–0.93)	0.009
Age (ref: <35 years)						
35–	N/A	N/A	N/A	N/A	0.884 (0.661–1.183)	0.408
45–	N/A	N/A	N/A	N/A	0.671 (0.49–0.918)	0.013
55–	N/A	N/A	N/A	N/A	0.842 (0.551–1.286)	0.426
Race (ref: Han)						
Minority	N/A	N/A	N/A	N/A	1.482 (0.985–2.23)	0.059

### 3.3 Relationship between WFC and OR of anxiety based on RCS

**Figure 2:**
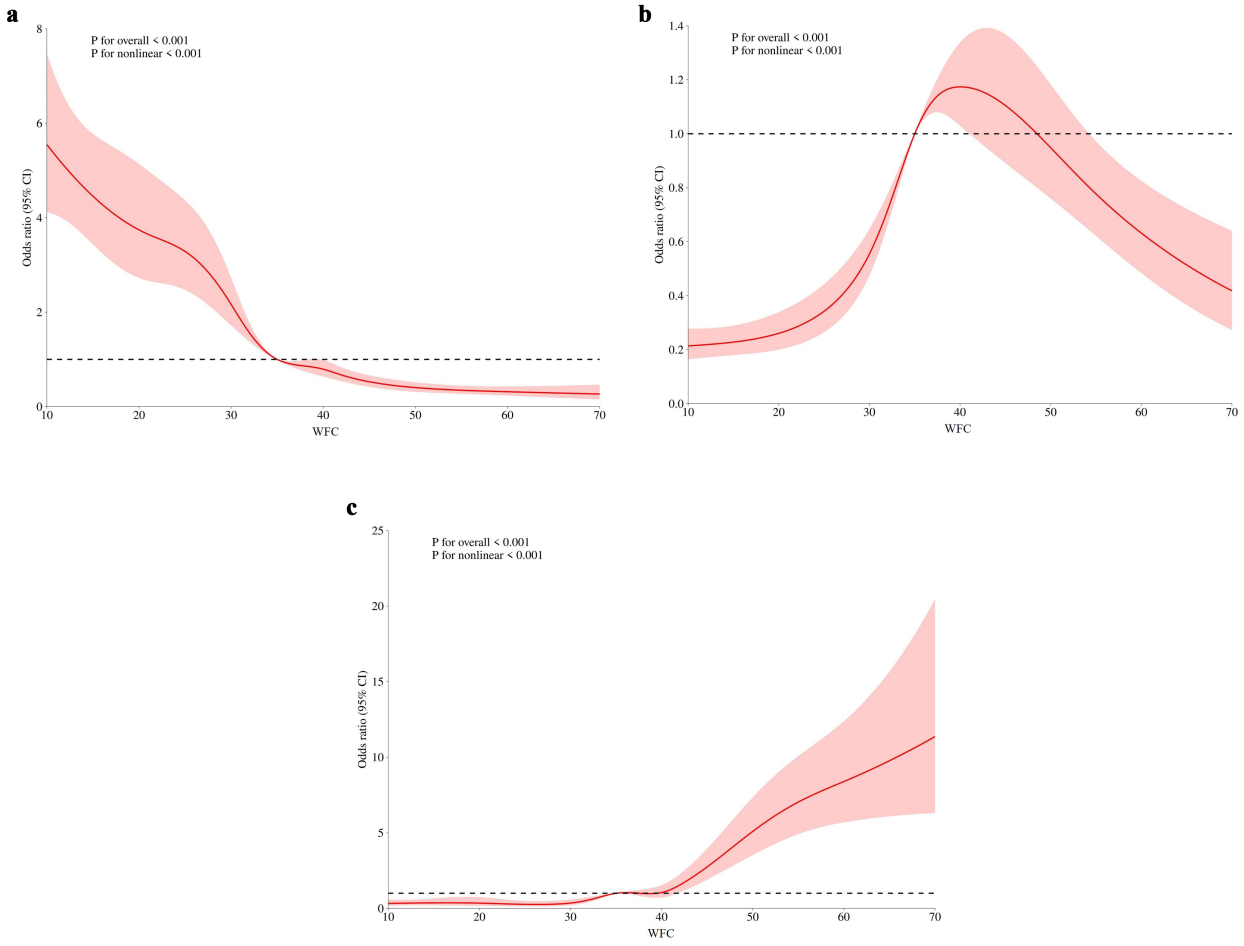
The relationship between WFC and OR of anxiety: **(A)** no anxiety, **(B)** mild anxiety, and **(C)** severe anxiety. WFC: work–family conflict.

As depicted in Figure 2, participants could remain anxiety-free when their WFC scores were below 35, suggesting that individuals were generally able to manage lower levels of conflicts effectively. However, as WFC scores increased beyond this threshold, the risk of experiencing anxiety rose significantly. Specifically, within the range of 35 to 40 points on the WFC, a notable increase in the odds of mild anxiety was observed. When WFC scores exceeded 40, the odds of moderate to severe anxiety increased substantially. The RCS analysis revealed a clear pattern: as WFC increased, the risk of anxiety correspondingly rose, with a particularly sharp escalation at higher levels of conflict.

### 3.4 Correlation and linear regression analysis between WFC, family health, and anxiety scores

The bivariate correlation analysis revealed a statistically significant correlation between WFC, family health, and anxiety (*p* < 0.001). WFC was positively correlated with anxiety (*r* = 0.453), while family health exhibited a negative correlation with anxiety (*r* = −0.323).

A linear mediation analysis was conducted to provide a comprehensive understanding of the relationship between WFC, family health, and anxiety. First, family health (M) was regressed on WFC (X), controlling for demographic variables such as gender, age, and income. The results revealed that WFC significantly predicted family health, with higher levels of WFC associated with lower family health scores (*a* = −0.136; *p* < 0.001). This finding suggests that increased WFC negatively impacts family health. Second, anxiety (Y) was regressed on both WFC (X) and family health (M), again controlling for demographic variables. The results demonstrated that family health exhibited a significant negative effect on anxiety (*b* = −0.143; *p* < 0.001), suggesting that better family health was associated with lower anxiety levels. In contrast, WFC exerted a significant positive effect on anxiety (*c’* = 0.136, *p* < 0.001), indicating that higher levels of WFC directly increased anxiety levels.

### 3.5 Mediating effect of family health on the relationship between WFC and anxiety

Model 4, a simple mediation model available in the SPSS macro developed by Hayes (2022), was employed to examine the mediating role of family health in the relationship between WFC and anxiety. The results are presented in Tables 3, 4. The negative predictive effect of WFC on family health was statistically significant (*β* = −0.2723, *t* = −20.1394; *p* < 0.001), thereby supporting hypothesis H1. Additionally, the negative predictive effect of family health on anxiety was statistically significant (*β* = −0.2157, *t* = −14.0457; *p* < 0.001), verifying hypothesis H2. The positive predictive effect of WFC on anxiety was statistically significant (*β* = 0.4532, *t* = 36.1957; *p* < 0.001), thereby supporting hypothesis H3. Upon inclusion of the mediating variables, the positive predictive effect of WFC on anxiety remained significant; however, the effect size was reduced (*β* = 0.3945, *t* = 31.688; *p* < 0.001). Furthermore, the upper and lower limits of the bootstrap 95% CI for the mediating effect of family health did not include 0 (Table 4), confirming that the mediating effect was present, which was partially mediated, thereby verifying hypothesis H4.

**Table 3:** Mediation model testing the role of family health.

Predictor variables	Anxiety			Family health			Anxiety		
	*β*	SE	*t*	*β*	SE	*t*	*β*	SE	*t*
WFC	0.4532**	0.0125	36.1957	−0.2723**	0.0135	−20.1394	0.3945**	0.0127	31.688
Family health							−0.2157**	0.0127	−17.0457
*R* ^2^	0.4533			0.2923			0.4986		
Adjustment *R*^2^	0.2055			0.0741			0.2486		
*F*-value	1310.125**			405.596**			837.782**		

**p < 0.01.

**Table 4:** Decomposition of total, indirect, and direct effects.

Effect	Pathway	Effect value	Effect proportion (%)	95% CI		*p*-value
				Lower	Upper	
Total effect		0.4532	100.00	0.4287	0.4778	<0.001
Direct effect	WFC → Anxiety	0.3945	87.05	0.3697	0.4193	<0.001
Indirect effect	WFC → Family health → Anxiety	0.0587	12.95	0.0498	0.0684	<0.05

**Figure 3:**
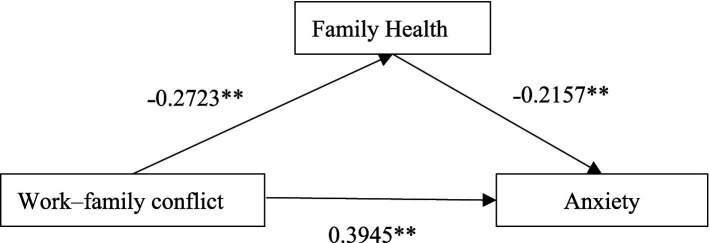
The mediating effect of family health on the relationship between WFC and anxiety (**p < 0.01).

The results indicated that WFC significantly and positively predicted anxiety, whereas family health significantly and negatively predicted anxiety. Moreover, family health was associated with anxiety through the mediating effect of WFC. The mediation model is presented in Figure 3.

### 3.6 Sensitivity analysis

In the sensitivity analysis, the numerical variable of anxiety scores was used as the dependent variable, and a stepwise multiple linear regression was employed. The initial independent variables included sex, age, race, family structure, marital status, number of children, family income, occupational status, number of diseases, medical insurance, family health, and WFC. The regression model was statistically significant (*F* = 120.927; *p* < 0.001). WFC and family health exhibited statistically significant effects on anxiety scores, with opposing directions (*p* < 0.001). Married individuals, males, and respondents aged over 45 exhibited lower anxiety scores. Conversely, an increased number of diseases and children, ethnic minorities, and those without medical insurance were associated with higher anxiety scores (Table 5).

**Table 5:** Multiple linear regression of WFC, other variables, and anxiety.

Variables	*β*	SE	*t*	*p*-value	95% CI lower	95% CI upper
Constant	5.314	0.448	11.851	0.000	4.435	6.193
WFC	0.134	0.004	32.101	0.000	0.126	0.142
Family health	−0.132	0.008	−15.779	0.000	−0.148	−0.115
Sickness number	0.696	0.086	8.113	0.000	0.527	0.864
Marital status (married)	−1.085	0.214	−5.068	0.000	−1.505	−0.665
Sex (male)	−0.496	0.105	−4.737	0.000	−0.701	−0.291
Child number	0.250	0.080	3.108	0.002	0.092	0.408
Race (minority)	0.527	0.221	2.384	0.017	0.094	0.960
Medical insurance (yes)	0.336	0.153	2.201	0.028	0.037	0.635
Age group (45 and above)	−0.242	0.111	−2.191	0.028	−0.459	−0.026

### 3.7 Subgroup analysis

Subgroup analyses by gender were performed to further explore the association between WFC and anxiety, as well as the mediating role of family health across different groups. Although the indirect effect of the pathway was slightly higher in females than in males, the mediating effects of family health were statistically significant in both groups (*p* < 0.001). Detailed results are presented in Table 6.

**Table 6:** Decomposition of total, indirect, and direct effects by gender subgroup.

Effect	Pathway	Effect value	Effect proportion (%)	95% CI		*p*-value
				Lower	Upper	
Female						
Total effect		0.4232	100.00	0.3895	0. 4,568	**<0.001**
Direct effect	WFC → Anxiety	0.3628	85.73	0.3285	0.39713	**<0.001**
Indirect effect	WFC → Family health→Anxiety	0.0604	14.27	0.0065	0.0732	**<0.05**
Male						
Total effect		0.4962	100.00	0.4603	0.5321	**<0.001**
Direct effect	WFC → Anxiety	0.4392	88.51	0.4033	0.4752	**<0.001**
Indirect effect	WFC → Family health→Anxiety	0.0569	11.49	0.0442	0.0711	**<0.05**

Separate regression analyses were also conducted for males and females to assess the differential impacts of WFC. The results indicated a significant negative association between WFC and family health for females (*β* = −0.562; *p* < 0.001), whereas for males, WFC exhibited a significant negative association with family health (*β* = −0.474; *p* < 0.001). These subgroup analyses further demonstrated that the effect of WFC on family health was more pronounced in females compared to males.

## 4 Discussion

This study presents a comprehensive analysis of the relationship between WFC and anxiety among working parents, emphasizing the mediating influence of family health. The findings indicate a significant association between WFC and anxiety levels. This outcome is consistent with the existing literature that conceptualizes work–family tension as a precursor to occupational stress, which may subsequently develop into anxiety (Moreira et al., 2019; Xu et al., 2023). Such conflict stems from the interference between professional and familial responsibilities, primarily due to the competing demands of these two domains (Wang et al., 2021; Carvalho and Chambel, 2016). Elevated work demands frequently intrude upon the time, energy, and emotional resources allocated to family life, generating imbalance and anxiety (Ghislieri et al., 2017).

The findings further highlight the considerable impact of demographic variables on anxiety levels among working parents. Female participants reported higher anxiety levels than their male counterparts, possibly due to the greater domestic burden and caregiving responsibilities they disproportionately carry (Shockley and Singla, 2011; Sullivan and Gershuny, 2018). Younger parents, particularly those under 35, exhibited higher anxiety levels, potentially due to the substantial energy required to balance childcare with professional obligations (Wang et al., 2023). Additionally, participants with lower household income, more children, and higher incidence of illnesses exhibited elevated anxiety levels as a result of financial stressors and limited access to supportive resources. These findings emphasize the importance of incorporating demographic considerations into developing targeted interventions aimed at alleviating anxiety among working parents. WFC arising from persistent pressures such as family poverty, long working hours, and excessive workload has been associated with increased stress levels and chronic health conditions among working parents (Annink et al., 2016). Several studies have linked an increased risk of work–family imbalance to poorer health outcomes, particularly in cases involving job insecurity from temporary employment or elevated job demands (Grawitch et al., 2010). COR theory (Hobfoll, 1989) offers a relevant framework, proposing that individuals evaluate the resources necessary to meet demands, which may ultimately influence how personal resources are allocated. In modern societies, the emphasis on personal achievement may contribute to increased work demands, while focusing on family harmony may give rise to distinct stressors and corresponding coping strategies.

However, the specific expression and intensity of this relationship between WFC and anxiety may differ across cultural contexts. Emerging Asia economies have experienced significant growth in the past decades. While this growth has contributed substantially to the global economy, it has also redirected individuals’ social and economic priorities toward work-related pursuits (Budhwar et al., 2016). In China and some other regions, cultural emphasis on both professional dedication and family responsibilities intensifies the impact of WFC on working parents (Lau et al., 2005). Traditional values such as collectivism in the workplace and family-oriented norms concept, including filial piety toward elders and caregiving for children, may exacerbate the pressure to succeed in professional and domestic domains, thereby increasing the potential for conflict (Le et al., 2020; Thein et al., 2010).

A key contribution of this study is the identification of family health as a mediating factor in the relationship between WFC and anxiety. Individuals with higher family health scores are less prone to experiencing anxiety, even when reporting higher levels of WFC. This protective effect of family health is paramount, as it suggests that fostering healthy family dynamics could effectively mitigate the mental health impact of WFC (Hjálmsdóttir and Bjarnadóttir, 2021). This finding is consistent with the COR theory, which asserts that individuals strive to protect and develop resources they perceive as valuable (Zheng et al., 2022). The family is widely recognized as a central emotional and practical support source. A healthy family environment, characterized by strong relationships, effective communication, and mutual support, can provide a refuge from occupational stress and essential emotional resources for managing anxiety (Murtorinne-Lahtinen et al., 2016).

The mediating role of family health in the association between WFC and anxiety is not confined to Chinese society; it is observed across various cultural contexts. Research involving Japanese employees identified that assistance from colleagues and family considerably alleviates depressive symptoms, emphasizing the universal importance of social connections in mitigating occupational stress (Omichi et al., 2022). Similarly, a study conducted in Portugal found that healthcare professionals with stronger family support experienced lower levels of anxiety and depression, even under intensified work-related pressures (Costa et al., 2023). A study conducted in the United States revealed that caregivers of patients with lower social support and more family conflict exhibited greater anxiety, indicating that family dynamics and the state of family health play a pivotal role in psychological well-being (Mitchell et al., 2022). in Europe, a study in Italy also discovered that anxiety symptoms were associated with impairment in both work and home management activities (Carmassi et al., 2021). These findings are consistent with the present study’s focus on family health as a vital protective factor against anxiety, suggesting that the beneficial role of family health transcends cultural boundaries.

The implications of these findings are instructive for occupational mental health interventions. The results highlight the necessity for addressing the interaction between professional and family domains when formulating strategies to enhance mental health in the occupational population. Previous research revealed that most employees prioritize more flexible working arrangements over material compensation (Wong and Chan, 2021). Recognizing the mediating role of family health enables the development of more precise and effective interventions that mitigate workplace stressors and strengthen familial support systems.

For employers, fostering a supportive work environment that recognizes the importance of family life is imperative. This objective can be achieved through flexible working hours, remote options, and family-oriented policies (Gatrell et al., 2013). Employers are also positioned to take a proactive role by providing resources and workshops designed to help employees manage stress and maintain a healthy work–family balance. Additionally, family-supportive supervisor training can equip managers with the competencies required to identify and address the work–family challenges encountered by their employees. Supervisors who receive training to enhance empathy and flexibility are better able to create a more supportive work environment, which has been associated with reductions in WFC. Flexible work arrangements, such as remote work options and adaptable scheduling, offer employees greater autonomy in managing their work-life balance. The rapid adoption of digital technologies has increased the feasibility of telecommuting, thereby enabling employees to manage their professional and domestic responsibilities more effectively (Belzunegui-Eraso and Erro-Garcés, 2020). For employees, developing personal strategies to manage WFC remains essential. Such strategies may involve effective time management, establishing boundaries between work and home life, and pursuing social support from colleagues, friends, and family members. Furthermore, engaging in self-care practices and prioritizing mental health is advantageous for individuals.

This study also emphasizes the imperative to address the gender dynamics in the context of mediating pathway. While WFC affects individuals across genders, women disproportionately bear the burden of family responsibilities, which may exacerbate conflicts. This finding is consistent with previous studies (Shockley and Singla, 2011; Sullivan and Gershuny, 2018). Consequently, targeted interventions aimed at mitigating gender inequality in the allocation of domestic labor are essential for reducing WFC among women (Petersen and Hyde, 2014). Such measures may include promoting shared parenting practices and fostering greater involvement of men in familial obligations.

This study has some limitations that warrant consideration. First, its cross-sectional design limits the capacity to establish causal inferences between WFC, family health, and anxiety. Longitudinal research is necessary to clarify temporal order and establish causal relationships among these variables. Second, all variables were assessed through self-administered questionnaires, which may introduce biases due to self-perception, social desirability, or memory-related inaccuracies, potentially compromising the validity of the findings. Third, the sample consisted exclusively of Chinese working parents, which may restrict the applicability of the results to other cultural or occupational contexts. Work–family dynamics and their influence on mental health vary considerably across cultural contexts and occupational domains. Integrating insights from diverse cultural environments highlights the global relevance of the study’s findings while recognizing the complex ways in which cultural factors shape the relationship between WFC, family health, and anxiety. Future research should explore these dynamics within varied cultural frameworks through comparative methodologies, enhancing a more comprehensive understanding of universal patterns and culture-specific characteristics. Such investigations will enhance the generalizability of the findings and support the development of culturally sensitive interventions to promote the well-being of working families on a global scale. Furthermore, intervention-based research can be conducted to identify effective strategies for improving family health and to examine how these strategies contribute to alleviating anxiety among working individuals. Future studies should consider conducting stratified analyses, such as investigating the relationship between WFC and anxiety across subgroups defined by age, occupational category, and family structures and assessing variations in the mediating role of family health. Such investigations can provide more specific strategies for implementing health promotion programs in the workplace.

## 5 Conclusion

The findings of this study provide a refined understanding of the relationship between WFC, family health, and anxiety. By elucidating the mediating role of family health, the present study contributes to the existing literature by highlighting the significance of family dynamics within the context of occupational mental health. The results highlight the need for a comprehensive approach that addresses the work environment and the well-being of the family unit in alleviating the impact of WFC on anxiety. In the Chinese cultural context, cultural norms such as collectivism, family roles, and work ethics may further influence how working parents perceive and cope with WFC and anxiety. Future research should incorporate cross-cultural comparative analyses to examine how these dynamics differ across diverse cultural frameworks. Moreover, culturally tailored interventions could be developed and implemented to more effectively support working parents in addressing these challenges. This study constitutes a call to action for researchers, policymakers, and employers to collaborate in creating more supportive ecosystems for individuals in the workplace.

## Data Availability

The raw data supporting the conclusions of this article will be made available by the authors, without undue reservation.

## Footnotes

^1^ https://www.wjx.cn/
